# Molecular Mechanisms of Autophagy Decline during Aging

**DOI:** 10.3390/cells13161364

**Published:** 2024-08-16

**Authors:** Shaun H. Y. Lim, Malene Hansen, Caroline Kumsta

**Affiliations:** 1 Graduate School of Biological Sciences, Sanford Burnham Prebys Medical Discovery Institute, 10901 North Torrey Pines Road, La Jolla, CA 92037, USA; 2 Program of Development, Aging and Regeneration, Sanford Burnham Prebys Medical Discovery Institute, 10901 North Torrey Pines Road, La Jolla, CA 92037, USA; mhansen@buckinstitute.org; 3 Buck Institute for Research on Aging, 8001 Redwood Boulevard, Novato, CA 94945, USA

**Keywords:** aging, autophagy, proteostasis, lysosomes, proteolysis

## Abstract

Macroautophagy (hereafter autophagy) is a cellular recycling process that degrades cytoplasmic components, such as protein aggregates and mitochondria, and is associated with longevity and health in multiple organisms. While mounting evidence supports that autophagy declines with age, the underlying molecular mechanisms remain unclear. Since autophagy is a complex, multistep process, orchestrated by more than 40 autophagy-related proteins with tissue-specific expression patterns and context-dependent regulation, it is challenging to determine how autophagy fails with age. In this review, we describe the individual steps of the autophagy process and summarize the age-dependent molecular changes reported to occur in specific steps of the pathway that could impact autophagy. Moreover, we describe how genetic manipulations of autophagy-related genes can affect lifespan and healthspan through studies in model organisms and age-related disease models. Understanding the age-related changes in each step of the autophagy process may prove useful in developing approaches to prevent autophagy decline and help combat a number of age-related diseases with dysregulated autophagy.

## 1. Introduction

Autophagy is a conserved multistep cellular recycling process that maintains cellular homeostasis and is associated with longevity in multiple organisms [1,2]. Reduced autophagy is associated with aging, while increased autophagy delays aging and extends lifespan in model systems including yeast, nematodes, flies, and mice [1,2]. Dysregulation of autophagy is associated with age-related diseases, including neurodegenerative disorders, cardiovascular diseases, and cancer [1,2]. Understanding how aging affects autophagy is essential for diagnosing and treating these age-related diseases.

**Figure 1:**
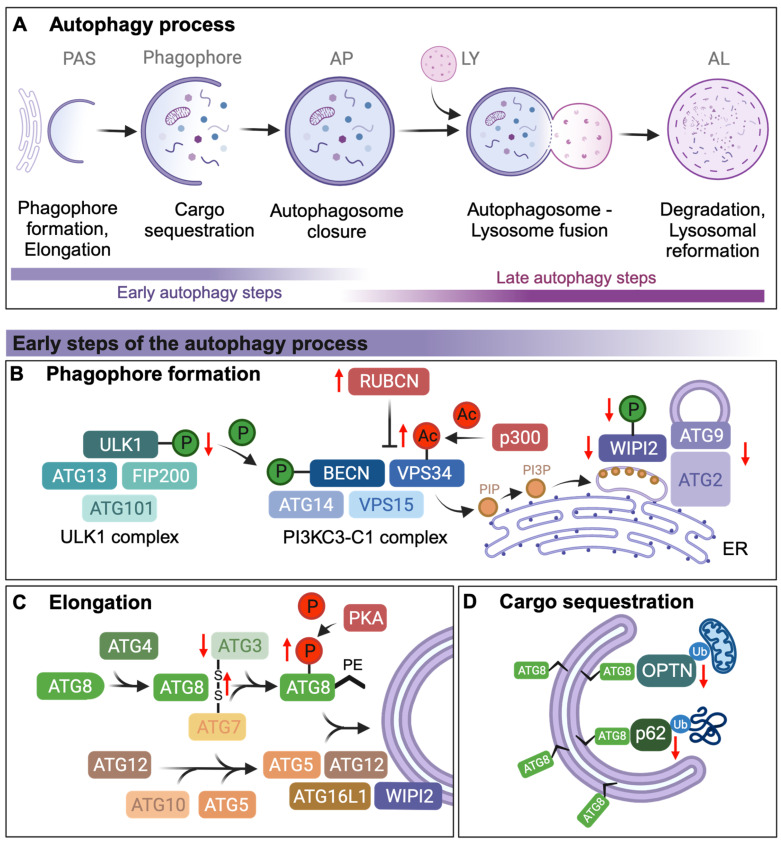
Age-related changes in the early steps of the autophagy process: (**A**) Overview of the autophagy process. Following initiation, the pre-autophagosomal structure (PAS) forms and elongates to form a phagophore. Autophagic cargo is sequestered to the PAS as it elongates and closes to become an autophagosome (AP). The AP subsequently closes fuses with the lysosome (LY) to form the autolysosome (AL). Autophagic cargo is subsequently degraded, while lysosomes reform from the AL. (**B**) Age-associated molecular changes in phagophore formation include decreased ULK1 phosphorylation (P), upregulation of RUBCN, increased acetylation (Ac) of VPS34 by p300, decreased WIPI2 protein levels and phosphorylation, and decreased *Atg2* and *Atg9* transcriptional levels. Phosphatidylinositol phosphate (PIP), Phosphatidylinositol 3-phosphate (PI3P), Endoplasmic reticulum (ER) (**C**) Age-associated molecular changes in phagophore elongation include loss of ATG3, increased intermolecular disulfide formation between ATG3 and ATG7, increased ATG8/LC3B Ser12 phosphorylation by PKA which prevents ATG8/LC3B conjugation to phosphatidylethanolamine (PE). ‘ATG8’ is used in the figure for simplicity. (**D**) Age-associated molecular changes in cargo sequestration include reduced levels of the autophagy receptors p62 and optineurin (OPTN). Ubiquitin (Ub). See Table 1 for a summary of age-related changes. Autophagy-inducing post-translational modifications are indicated in green while autophagy-inhibiting modifications are indicated in red. Red arrows denote age-related changes. See text for mechanistic details of the autophagy steps.

During autophagy, cellular components, called cargo, are delivered for recycling into lysosomes. Three distinct types of autophagy can be distinguished by how cargo is delivered to the lysosome; macroautophagy (via double-membrane autophagosomes), microautophagy (direct invagination of cargo into lysosomes), and chaperone-mediated autophagy (chaperone-mediated translocation of cargo into lysosomes via LAMP2 receptors) [1,2]. This review focuses on macroautophagy (hereafter termed autophagy), which refers to the sequestration of cargo into double-membrane structures known as phagophores that grow to become vesicles called autophagosomes. Autophagosomes fuse with lysosomes containing acidic hydrolases to form autolysosomes. The sequestered cargo and the inner membrane of the autolysosome are subsequently degraded and recycled to provide new building blocks for the cell [1,2] (Figure 1A). 

Multiple lines of evidence from animal models, including *C. elegans*, *Drosophila*, mice, and human tissues, as well as postmortem brains and induced neurons from neurodegenerative disease patients, suggest that autophagy declines with age. Age-related changes in upstream autophagy regulators, such as mTORC1 and AMPK, impact nutrient and energy-sensing pathways and can contribute to the decline in autophagy initiation with age [3]. Transcriptional regulators of autophagy, such as TFEB and FOXO, which are regulated by these upstream pathways, may also contribute to the age-related decline of autophagy, since age-related transcriptional decreases in select autophagy-related genes have been observed in various organisms, including *C. elegans* [4,5], *Drosophila* [6], mouse macrophages [7], human peripheral blood cells [8], and in aged human brains [9]. Likewise, select autophagy proteins decline with age in *Drosophila* [6], mouse brains [10], and in human osteoarthritic chondrocytes [11]. The accumulation of autophagosomes and aberrant autophagic structures in *C. elegans* [12], mouse motor neurons [13,14], human osteoarthritic chondrocytes [11], and induced neurons from Parkinson-disease patients [15] indicate age-related changes likely in late steps of the autophagy process. Autophagy flux assays, which track the dynamic changes to autophagosome numbers using inhibitors that block the final steps of autophagy, confirm a block in the autolysosomal degradation in aged *C. elegans* [12] and in senescent human fibroblasts [10]. 

These collective data indicate that autophagy dysfunction can occur at different steps of the autophagy process, but the specific molecular mechanisms driving autophagy decline in different models and tissues remain unclear [1]. This review specifically focuses on the effects of aging on the molecular steps of autophagy that are mediated by conserved autophagy-related (ATG) proteins. We discuss age-related changes in the autophagy process in the following sections: (1) phagophore formation, comprising initiation, double-membrane nucleation, and phagophore elongation; (2) cargo sequestration; (3) autophagosomal closure, lysosome maturation, autophagosome–lysosome positioning, and lysosome-autophagosome fusion; and (4) cargo degradation in the autolysosome and autophagic lysosome reformation [1,2]. For the purposes of this review, standard protein and gene nomenclature are used according to the organism discussed (e.g., human, rat, mouse, *Drosophila, C. elegans,* or yeast). We summarize the key observations in Table 1, highlighting how aging affects the expression and function of autophagy proteins and the post-translational modifications of autophagy proteins and leads to the accumulation of aberrant autophagosomal structures, consistent with stalled autophagy and altered cargo degradation in the autolysosome. 

Given the critical role of autophagy in determining healthspan and lifespan, understanding how age-related changes affect the individual steps of the autophagic degradation process is essential. This knowledge will enhance our ability to diagnose and treat age-related diseases associated with autophagy dysfunction. 

## 2. Autophagosome Formation and Elongation

Autophagosome formation is initiated by the ULK1 complex, which comprises ULK1, the scaffold protein FIP200, and the regulatory subunits ATG13 and ATG101. Since ULK1 complex phosphorylation is an initial step of autophagosome formation, age-related changes in the phosphorylation status of ULK1 could have profound effects on the ability to initiate autophagy. Indeed, the activating phosphorylation of Ser555 of ULK1 declines with age in human and murine skeletal muscle, but whether this age-related change is causative of an age-related decline of autophagy initiation has yet to be defined [16] (Figure 1B).

The activated ULK1 complex phosphorylates members of the PI3KC3-C1 complex, which consists of the regulatory subunit Beclin1 (BECN1), the lipid kinase VPS34, VPS15, and ATG14. ULK1 directly phosphorylates BECN1 of the PI3PC3-C1 complex on Ser14, which leads to the activation of VPS34 [32]. Activated VPS34 then phosphorylates inositol membrane lipids to generate a pool of PI3P (phosphatidylinositol 3-phosphate) [33]. Acetylation of VPS34 by the histone acetyltransferase p300 inhibits its interaction with ULK1 and its phosphatidylinositol (PI) substrate, leading to a reduction in autophagy [34] (Figure 1B). Targeting p300 with its inhibitor nordihydroguaiaretic acid induces autophagy by promoting autophagosome formation in human cells and *C. elegans* [35] and extends lifespan in various model organisms, including *C. elegans* [35], mice [36,37,38], *Drosophila* [39], and mosquitos [40]. Thus, an increase in p300 levels during aging could lead to an increase in VPS34 acetylation, reducing its activity by inhibiting its interaction with ULK1 and thus inhibiting overall autophagy, although this remains to be directly tested.

BECN1, and thus autophagosome formation, can be inhibited via its Thr108 phosphorylation mediated by the Hippo kinase STK4, also known as MST1, in mouse cardiomyocytes [41]. While the effect of BECN1 inhibition via STK4/MST1 during aging is not known, *Stk4/Mst1* mRNA expression levels are differentially affected by age in different murine tissues, i.e., it is reduced with age in the heart, striatum, and liver but increased in the spleen [18]. Additionally, in the cortex and hippocampus, STK4/MST1 levels increase until midlife before decreasing with age [18]. Besides inhibiting BECN1, STK4/MST1 can also phosphorylate LC3B. LC3B is one of six mammalian ATG8 protein family members, namely LC3A, LC3B, LC3C, GABARAP, GABARAPL1, and GABARAPL2/GATE16, which are required for autophagosome biogenesis, cargo recruitment, and autophagosome–lysosomal fusion. ATG8 proteins are widely used as markers of autophagosomes due to their conjugation with pre-autophagosomal and autophagosomal membranes [42]. Inhibiting the phosphorylation of LC3B/ATG8 at Thr50 by STK4/MST1 prevents the directional transport of autophagic vesicles and facilitates the transport of autophagosomes to lysosomes for subsequent fusion [43,44]. This is in line with studies in *C. elegans*, where the loss of STK4/MST1 ortholog CST-1 shortens lifespan, while the overexpression of CST-1 extends *C. elegans* lifespan [45]. This dual regulatory and context-dependent role indicates that STK4/MST1 can both inhibit and promote different stages of autophagy, depending on the specific targets involved. It is thus possible that STK4/MST1 dysregulation during aging contributes to tissue-specific variability in the decline of autophagy. 

Rubicon (RUBCN), a BECN1-interacting protein, directly binds and inhibits VPS34 lipid kinase activity, which disrupts autophagosome formation [46,47]. RUBCN is increased at both transcriptional and translational levels with age in *C. elegans*, *Drosophila*, and mouse tissues and is associated with impaired autophagy during aging [48] and may thus contribute to the overall decline in autophagy with age by decreasing VPS34 activity and reducing PI3P levels (Figure 1B). Notably, PI3P levels are selectively decreased in the brain of patients with Alzheimer’s disease (AD) and AD mouse models [49], which could contribute to autophagy inhibition in AD [50]. 

Once the PI3KC3-C1 complex generates the PI3P pool on the primary autophagosome-related membrane, the phagophore recruits WIPI2 via its PI3P-binding domains, which subsequently forms a complex with ATG2 and ATG9 to establish phagophore-ER contact sites [51]. Lipids are delivered to the growing vesicles by ATG2, a lipid-transfer protein, which transfers phospholipids from the ER and possibly other membrane sources, which include the Golgi apparatus and ATG9-positive vesicles [52], which then fuse with the growing phagophore during elongation [53]. *ATG2* knockout studies in mammalian cells have shown inhibited membrane expansion [54,55], while partially formed autophagic structures have been observed in *Drosophila* and *C. elegans* deficient for *Atg2* [56,57]. Furthermore, *Atg2* transcript levels have also been found to be significantly decreased with age in *Drosophila* [6]. The loss of *Atg2, Atg18,* and *Atg9* transcripts in *Drosophila* heart and flight muscles via tissue-specific RNAi knockdown significantly decreases healthspan and lifespan [58] (Figure 1B). A recent study of aged mouse hearts discovered that ATG9 protein and transcript levels decline with age leading to reduced autophagosome formation, compromising overall autophagy [59] (Figure 1B). It is possible that the reduction in the machinery that coordinates phagophore elongation could contribute to autophagy decline with age.

Similarly, loss of WIPI2 in human cell models prevents direct lipid transport leading to an accumulation of ATG9 at forming autophagosomes [60]. This in turn may prolong the association of ATG2 with the phagophore, which could potentially enable unregulated lipid transfer and contribute to the formation of multilamellar structures that are not able to develop into LC3B/ATG8-positive autophagosomes, as previously described in mice neurons and also seen in the brains of AD patients [14]. Indeed, autophagosome formation seems to stall at the elongation process in isolated ganglion neurons from aged mice due to loss of WIPI2 levels and/or phosphorylation (Figure 1B). Increasing the levels of WIPI2B in these aged neurons is sufficient to restore autophagosome formation [14], indicating that WIPI2 levels and/or phosphorylation are critical for maintaining autophagy with age, at least in ganglion neurons. 

Phagophore elongation is mediated by two ubiquitin-like conjugation systems, the ATG8/LC3 and ATG12 systems, which conjugate ATG8 family proteins with phagophore-resident lipid phosphatidylethanolamine (PE) in a process known as lipidation [42]. One way in which phagophore elongation may decline with age is via decreased LC3B/ATG8 lipidation. For instance, defective LC3B/ATG8 lipid conjugation in *Atg3* knockout mice leads to autophagosome malformation [61]. LC3B/ATG8 lipidation is also decreased in the aortas of aged mice, where an age-related increase in oxidative stress correlates with an increase in disulfide bonds between ATG7 and ATG3. Supporting the hypothesis that oxidative stress inhibits autophagy at the level of autophagosome elongation, H_2_O_2_ treatment prevents LC3B/ATG8 lipidation in HEK and rat aortic smooth muscle cells. This inhibition is due to the direct oxidation of ATG3 and ATG7, leading to intermolecular disulfide formation between ATG3 and ATG7 or stable S-glutathionylation of ATG3 or ATG7 [19] (Figure 1C). Furthermore, the phosphorylation of lipidated and unlipidated LC3B/ATG8 at Ser12 by protein kinase A (PKA) in human neuroblastoma cells reduces rapamycin-induced recruitment of LC3B/ATG8 to the autophagosome [20] (Figure 1C). Given prior studies showing that increased PKA activity is detrimental to healthspan [62] and that inhibition of PKA activity increases lifespan and promotes healthy aging [63,64], it is possible that the dysregulation of PKA activity during aging may lead to aberrant LC3B/ATG8 Ser12 phosphorylation that reduces overall autophagy by preventing autophagosome elongation. Interestingly, overexpression of ATG5, which is essential for autophagy activation via LC3B/ATG8 lipidation and ATG12-ATG5 conjugation, has been shown to extend lifespan in mice [65]. The changes in ATG5 levels and function with age remain to be explored to determine if functional decline in ATG5 is another potential mechanism contributing to age-associated autophagy reduction. However, it should be noted that autophagy-related proteins that help conjugate ATG8-family proteins to membranes, such as ATG5 and ATG16L1, may also perform noncanonical functions. These include alternative vesicular pathways like LC3-associated phagocytosis and LC3-associated endocytosis, as well as unconventional secretory pathways involving the formation of extracellular vesicles. While the role of these noncanonical autophagy pathways in aging and age-related diseases remains to be fully elucidated (reviewed in [42,66]), a recent study of *C. elegans* showed that reduced levels of autophagy genes involved in ATG8/LC3 conjugation lead to the formation of so-called exophers, which are large neuronal extrusions [67,68].

## 3. Cargo Sequestration via Autophagy Receptors

During cargo sequestration, cytoplasmic material, including ubiquitinated protein aggregates (aggrephagy) and damaged mitochondria (mitophagy), are sequestered in growing phagophore. For detailed reviews on these selective autophagy pathways and their deregulation, the reader is referred to [69,70]. Here, we discuss how autophagy receptor proteins, which mediate selective cargo sequestration by binding to ATG8-family proteins via LC3-interacting regions (LIRs), change with age and affect aging-related processes. Autophagy receptors facilitate the interaction between their respective cargo and the phagophore and are themselves degraded during autophagy.

The metazoan autophagy receptor p62/SQSTM1 binds to ubiquitinated cargo, such as aggregated proteins and damaged mitochondria via its UBA domain. *p62/SQSTM1* (Ref(2)P in *Drosophila*) transcript levels decrease with age in *Drosophila* and in mouse models [21,22]. Age-correlated oxidative damage at the *p62* promoter in humans reduces *p62* transcript levels and is associated with the onset of neurodegenerative diseases [71,72] (Figure 1D). Interestingly, overexpression of p62/SQST-1 or Ref(2)P has been shown to extend lifespan via autophagy and mitophagy induction in *C. elegans* and *Drosophila,* respectively [21,73]. Therefore, it is possible that a decline in p62 contributes to autophagy decline with age by preventing cargo sequestration and subsequent accumulation of autophagic cargo, as well as via reduced autophagy induction. Similarly, another recent study found that upregulation of the outer mitochondrial membrane protein NIX/BNIP3L, which also functions as a mitophagy receptor [74], prevents the accumulation of damaged mitochondria in aging *Drosophila* brains [74], further suggesting a role for autophagic receptors in preventing autophagic decline during aging. 

Another mitophagy receptor, optineurin (OPTN), has been implicated in the clearance of damaged mitochondria by autophagy via binding to LC3/ATG8 [75]. Mutations in OPTN disrupt the autophagosomal recruitment of damaged mitochondria and are causative of the neurodegenerative disease amyotrophic lateral sclerosis [75]. OPTN translocation to the mitochondria and mitochondrial clearance is disrupted by age-related cholesterol accumulation in aging mice [23] (Figure 1D). 

Thus, the decline in levels of autophagy receptors such as p62 and OPTN with age can impair the sequestration and degradation of cellular cargo, contributing to autophagic decline and age-related diseases. However, additional work is required to identify and validate additional autophagy receptors and explore how the loss of these receptors during aging could affect overall autophagy as well as cargo degradation. See Figure 1 and Table 1 for a summary of how early autophagy steps may decline with age. In the next section of the review, we explore how late autophagy steps may decline with age.

## 4. Autophagosomal Closure and Autophagosomal Lysosomal Fusion

**Figure 2:**
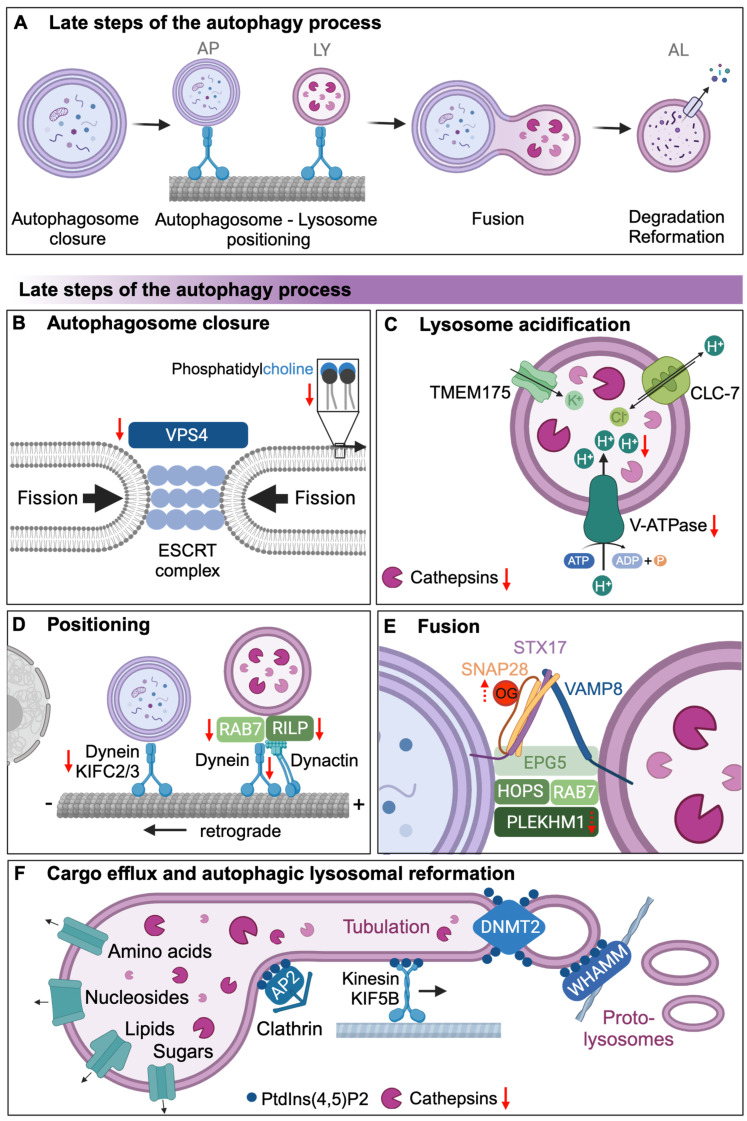
Age-related changes in the late steps of the autophagy process: (**A**) Overview of the late steps of the autophagy process. Following closure of the phagophore, the autophagosome (AP) and mature lysosomes (LY) are transported into close proximity for subsequent fusion to form autolysosomes (AL). In the AL, cargo is degraded and transported back into the cytosol before autophagic lysosomal reformation occurs. See text for mechanistic details of the late autophagy steps. (**B**) Age-associated molecular changes in autophagosomal closure include decreased phosphatidylcholine levels and ESCRT complex component VPS4 protein levels. (**C**) Age-associated molecular changes in lysosomal acidification include reduced levels of V-ATPase components, which lead to increased pH levels and decreased cathepsin activity. (**D**) Age-associated molecular changes in AL-AP positioning include a decline in motor proteins dynein and KIFC2/3, as well as adaptor proteins RAB7 and RILP, and decreased interaction between dynein and dynactin. (**E**) Age-associated molecular changes in AL-AP fusion include reduction in PLEKHM1 and potentially increased O-GlcNAc (OG) modification of SNARE protein SNAP-29. (**F**) Age-associated molecular changes in metabolite efflux could be influenced by decreased cathepsin activity, while no age-related changes in autophagic lysosomal reformation (ALR) are known. See Table 1 for a summary of age-related changes. Red arrows denote age-related changes; dashed red arrow indicates potential age-related changes. See the text for mechanistic details of the autophagy steps.

Autophagosomal closure and lysosomal maturation are required for the late steps of the autophagy process (Figure 2A). For autophagosomal closure, two membrane-merging events are required: the double-membraned ends of the forming autophagosome must undergo membrane fission to first form separate inner and outer membranes, which then fuse to form a closed autophagosome. ESCRT proteins are essential for membrane fission (Figure 2B) as ESCRT dysfunction is associated with phagophore accumulation, as observed following loss of ESCRT-III complex components in mouse cortical neurons and *Drosophila* [76]. Membrane fission also requires the AAA+ ATPase VPS4, which together with the ESCRT-III complex generates an ATP-dependent force for fission [77,78]. During yeast aging, the protein abundance of Vps4 sharply decreases despite stable transcript levels [24], suggesting decreased translation or increased turnover of Vps4, which may play a key role in preventing membrane fission and subsequent autophagosomal closure at least in aging yeast (Figure 2B). It remains to be explored, however, how the ESCRT complex components decline which age, which may further inhibit membrane fission and autophagosomal closure.

Autophagosomal closure is also affected by the lipid composition of the autophagosomal membrane. Loss of the most abundant lipid in the autophagosome membrane, phosphatidylcholine (PC), impairs the closure of autophagosomes in yeast [79]. Inhibition of de novo PC synthesis significantly decreases autophagic processing, leading to the accumulation of open cup-like structures [79]. A reduction in overall PC contents has been reported with age in several species and tissues (Figure 2B), while the aging rat brain seems to accumulate PC [80]. However, studies directly linking age-related changes in PC levels and their effect on autophagy still have to be conducted.

Mature lysosomes contain active hydrolases, enzymes that catalyze the hydrolysis of autophagic cargo. These hydrolytic enzymes are active at acidic pH levels (~pH 4–5), which is maintained in the lysosome by the coordinated activity of the proton pump V-type ATPase, the Cl^−^/H^+^ antiporter ClC-7, and the K^+^ cation channel TMEM175 [81]. The ClC-7 antiporter and K^+^ cation channel maintain transmembrane voltage by Cl^−^ influx or K^+^ efflux, respectively, when protons are pumped into the lysosomal lumen by the V-ATPase. Vacuolar acidity declines with age in yeast [29] (Figure 2C), and lysosomal pH increases in aging *C. elegans* intestines [30]. The lysosomal degradation activity in *C. elegans* declines during aging [82], which could be due to the diminished expression of some of the ~40 lysosomal genes, including cathepsin proteases and V-ATPase subunits [4]. Long-lived *C. elegans daf-2*/insulin/IGF-1 receptor mutants maintain lysosomal gene expression and lysosomal pH levels with age [4]. Consistently, in yeast, the overexpression of V-ATPase components Vma1 and Vph2 prevents the vacuolar pH increase with age and extends lifespan [29]. A recent study of *C. elegans* muscle tissues identified a microRNA, *mir-1*, which negatively regulates lysosomal V-ATPase activity by directly controlling protein expression of VHA-13/ATP6V1A. Accumulation of *mir-1* with age could reduce lysosomal activity by decreasing V-ATPase components [83], at least in *C. elegans* muscle. This suggests that factors that regulate V-ATPase activity or assembly may thus regulate V-ATPase activity during aging, although this has been largely unexplored. Interestingly, cultured neurons from mice with deletions in the ClC-7 H^+^/Cl^−^ antiporter display no changes in lysosomal pH, but the mice suffer from neurodegeneration and lysosomal storage diseases, indicating that disturbing lysosomal ion transport can affect lysosome function independently of changing the pH [84]. Additionally, lysosomal damage during aging may further contribute to the decline in autophagosomal degradation in mice and humans [84]. For example, lysosomal membrane permeabilization, which is the loss of lysosomal membrane integrity caused by various forms of stress, exacerbates overall lysosomal dysfunction and leads to a subsequent decline in autophagy [85]. 

Before the fully closed autophagosomes can fuse with acidic lysosomes, autophagosomes and lysosomes have to come into close proximity. The distribution of lysosomes in the cell can be influenced by the abundance of nutrients. Starvation results in the perinuclear localization of lysosomes (retrograde transport) in HeLa cells [86], while lysosomes move towards the cell periphery (anterograde transport) upon an abundance of nutrients or during growth factor signaling [86]. Transport of autophagosomes and lysosomes to the perinuclear region is achieved by retrograde transport mediated by motor proteins on the microtubule network [87]. Specifically, retrograde transport is achieved by minus-end-directed motor proteins (dynein and members of the *C-kinesin* family such as KIFC2 and KIFC3) [88], but as cells age, their ability to move autophagosomes and lysosomes closer to the cell center decreases, contributing to less efficient fusion and cargo degradation [88] (Figure 2D). This is attributed to a diminished interaction of autophagosomes and lysosomes with the motor proteins dynein and KIFC3, as observed in hepatocytes from old mice [88]. Lysosomes interact with dynein complexes via dynactin, RAB7 and its effector protein RILP. The dynein interaction with dynactin declines with age (Figure 2D), thus impairing dynein function, which contributes to Aβ pathology in cynomolgus monkey brains [27,28]. Moreover, transcriptional profiling analysis of dendritic cells isolated from human peripheral blood mononuclear cells showed reduced expression of *RILP* in aged compared with young individuals [25]. While reduced *RILP* expression may affect antigen-presenting capacity in dendritic cells, it may also potentially inhibit lysosome positioning, reducing overall autophagic activity. Interestingly, decreased RAB7 levels during ovarian aging leads to autophagosome formation defects and accumulation of damaged mitochondria from decreased mitophagy in oocytes, implicating RAB7 as an additional player in maintaining mitochondrial homeostasis during aging [89]. However, the implications of the loss of both RILP and RAB7 activity in the larger context of organismal aging and autophagy decline have yet to be studied in greater detail.

Autophagosome–lysosome fusion is modulated by two SNARE complexes. The Q-SNARE complex (STX17-SNAP29) is recruited to the autophagosomes and the R-SNARE complex (VAMP7/8 or YKT6) is lysosome-associated [90]. Tethering of autophagosomes and lysosomes is mediated by EPG5, a scaffold protein that binds ATG8 family proteins and Rab7, stabilizing the STX17-SNAP29-VAMP8 SNARE complex [87], and another tethering factor PLEKHM1, which forms a complex with RAB7 and VPS41 of the HOPS complex that tethers autophagosomes and lysosomes, SNARE complexes, and membrane-bound ATG8. Loss of PLEKHM1 has been shown to inhibit autophagosome–lysosome fusion [91] (Figure 2E), but to date, little work has been conducted investigating how autophagosome–lysosome fusion changes with age and how this may affect overall autophagic rates. Interestingly, in *C. elegans*, the SNARE protein SNAP-29 has been shown to be post-translationally modified by O-linked β-N-acetylglucosamine (O-GlcNAc) transferase (OGT), which negatively regulates autophagy [92], and it has been shown that O-GlcNAc levels are increased during aging in rats [93]. It remains to be investigated how post-translational modifications (e.g., dephosphorylation of STX17, or O-GlcNAcylation of SNAP-29), dysregulation of tethering factors and SNARE protein levels, and disruptions in the assembly of the relevant complexes (e.g., HOPS complexes) are affected by aging to determine their role in autophagic decline with age (Figure 2E).

## 5. Lysosomal Degradation of Autophagic Cargo and Autophagic Lysosomal Reformation

Various types of cargo, such as lipids, DNA, RNA, and proteins, are degraded in the lysosome by acidic hydrolases [94]. Autophagic protein degradation is mediated by the most abundant lysosomal proteases, known as cathepsins [95], which are classified according to the amino acids in their catalytic active sites (i.e., serine, aspartyl, and cysteine), with cysteine and aspartyl proteases representing most lysosomal activity [95]. Cathepsins have varying optimum pH levels, and given that lysosomal pH increases with age in yeast [29] and *C. elegans* [4,30], cathepsins may not be functioning in an optimal environment in aging animals, thus affecting overall lysosomal degradation activity (Figure 2C). Accordingly, cathepsin activity declines with age, as measured by a decline in mature cathepsin L in older *C. elegans* [4]. In aging rat urothelial cell lysates, cathepsin B activity was significantly decreased with age, while endolysosomal pH (~pH 6.0) was higher in old urothelia. Aberrantly large endosomes were also observed in rat urothelia in the same study, which suggests that lysosomes are fusing with endosomes but that degradation is impaired, likely due to the age-associated changes in lysosomal pH [31]. Interestingly, mature cathepsin B is expressed higher in aging rat livers [96]. These findings suggest that autolysosomal degradation via cathepsin activity may be regulated on several levels, which include downregulated cathepsin proteins, increased pH levels, or an increase in endogenous cathepsin inhibitors, such as serpins and cystatins, although further work is required to directly show this. Lysosomal hydrolases can function as lifespan-regulating signals, since the increased intestinal expression of the lysosomal lipase LIPL-4 in *C. elegans* increased longevity via gene expression changes caused by a lysosome-to-nucleus signaling pathway [97]. In germline-less *C. elegans* mutants, which are long-lived, LIPL-4 was suggested to mediate longevity via the increased autolysosomal degradation of fat [98]. However, it remains unclear how LIPL-4 levels are regulated during aging. Additionally, more work is required to further examine how the degradation of additional autolysosomal cargo such as sugars, DNA, and RNA declines with age and could affect health and longevity.

After the degradation of autolysosomal cargo, metabolites, including amino acids, sugars, lipids, and nucleosides are either stored in the lysosome or returned to the cytosol via transport proteins [99] (Figure 2F). A recent study identified many lysosomal transport proteins by the isolation of lysosomal membranes from rat liver cells, but for many, neither the substrate nor their regulation has been confirmed [100]. While mutations in lysosomal transport proteins can lead to lysosomal storage disorders and neurodegeneration, the effect of aging on the integrity and function of lysosomal transport proteins is not known and requires further investigation. 

The final step of autophagy involves autophagic lysosome reformation (ALR) [101]. Here, proto-lysosomes, which are small vesicles consisting of lysosomal membrane components, extend from autolysosomes, bud off, and mature into functional lysosomes to renew the lysosome pool [101] (Figure 2F). This is achieved through the generation and regulation of membrane-bound phosphoinositides, particularly PtdIns3P, PtdIns4P, and PtdIns(4,5)P2. These phosphoinositides recruit specific effector proteins that drive membrane remodeling [102]. PtdIns(4,5)P2 recruits the adaptor protein-2 (AP-2)-clathrin complex, forming a clathrin lattice that stimulates membrane budding. The kinesin motor protein KIF5B, which binds to PtdIns(4,5)P2, “pulls” membrane buds along microtubules to form reformation tubules, while actin polymerization driven by the PtdIns(4,5)P2-binding protein WHAMM aids in membrane extrusion. Dynamin 2 (DNM2), a large GTPase protein that also binds to PtdIns(4,5)P2, is critical for the scission of reformation tubules. PtdIns3P is essential for the final stage of ALR, where it helps facilitate the cutting and separation of reformation tubules from autolysosomes. Since dysregulation of these phosphoinositide-dependent processes inhibits ALR and leads to the depletion of lysosomes and autophagy inhibition [101], age-related changes could have profound effects on ALR and lysosome abundance, as well as autophagy flux. However, little is known about the changing dynamics of lysosome reformation and the proteins that govern this process during aging. While clathrin levels are increased in the frontal cortex of men aged 70–90 compared with younger individuals aged 20–55 [103], the physiological consequences of this increase on ALR or other clathrin-mediated processes are yet to be identified. Recent findings in *C. elegans* have highlighted the role of the fatty acid elongase ELO-3 in regulating ALR through the biosynthesis of glucosylceramide (GlcCer), a crucial component of intracellular membranes. Reduced ELO-3 activity impairs the levels of GlcCer, which disrupts clathrin recruitment to autolysosomes, thus inhibiting ALR and leading to enlarged autolysosomes, increased mTORC1 activation, and reduced lifespan [104]. This suggests that age-related decline in lipid metabolic pathways could impair ALR, contributing to decreased autophagy with age. Another recent study in *C. elegans* described the increased formation of autophagic tubular lysosomes following lifespan-extending dietary restriction and found that the constitutive induction of tubular lysosomes is sufficient to improve healthspan, albeit without extending lifespan [105]. It is, however, not yet clear whether these tubular lysosomes are part of ALR. Understanding how aging affects ALR and its regulation is crucial, as age-related changes in lipid metabolism and phosphoinositide signaling could significantly impact autophagic efficiency and cellular health. See Figure 2 and Table 1 for a summary of how late autophagy steps may decline with age.

## 6. Conclusions

While a decline in autophagy with age has been documented in multiple organisms, research on how aging affects the individual steps of autophagy is still underway. Age-related changes in the levels of bona fide autophagy machinery proteins functioning in several autophagy-regulating steps, as well as post-translational modifications that control the activity of autophagy proteins, become dysregulated with age and likely contribute to the overall decline in autophagy (Table 1). It is unclear which molecular step(s) in the autophagy process drive systemic autophagy changes with age, although an increase in autophagosome numbers and reduced flux with age suggest stalled late-autophagy steps [12]. In particular, it remains unclear how a combination of multiple points of failure in autophagy with age drives the decline of the overall process during aging. Mechanistic knowledge of the individual steps of autophagy allows for potential modulation to boost autophagy in instances of age-associated decline to help promote proteostasis, although it should be noted that the mechanisms for autophagy decline could be different across various tissues. Different rates of autophagy decline with age have been observed in distinct tissues in *C. elegans*, *Drosophila*, murine, and human cell models [1,12,106]. Additional work is clearly required to investigate the molecular dynamics of such decline. As inhibition of early-acting autophagy genes involved in autophagy initiation in *C. elegans* neurons causes expulsion of cellular cargo in secretory vesicles [67,68], reduced autophagic degradation may lead to increased noncanonical functions of autophagy proteins. Whether stalled autophagy in one tissue affects autophagy and proteostasis in other tissues via inter-tissue communication still has to be investigated. Furthermore, it should be reiterated that many autophagy components are also required in additional cellular processes. This is true for essentially all late-acting autophagy genes involved in fusion, degradation, and closure, e.g., SNARE complexes are involved in the fusion of other types of vesicles, such as synaptic vesicles. It is thus crucial to carefully consider how to target components that are specific to autophagy that would not affect other processes to effectively improve human healthspan by boosting autophagy.

While the molecular mechanisms of both aging and autophagy have been studied in significant detail, additional research is required to integrate these two fields, especially in a temporal and spatial manner, as well as in response to interventions. Much work has already been carried out to investigate the consequences of modulating autophagy in model organisms or disease models, but whether these findings translate to aged humans remains unclear and is an important area for future research.

## Figures and Tables

**Table 1:** Summary of age-related changes in the individual steps of autophagy.

Step	Individual Step	Molecular Mechanism	Model System	Reference
1	Autophagosome formation and elongation	Decline in Atg2, Atg5, and Atg7 transcriptional levels	Human, *Drosophila*	[6,9]
		Decreased ULK1 Ser555 phosphorylation	Mouse	[16]
		VPS34 acetylation by increased p300	Human	[17]
		BECN-1 inhibition by RUBCN upregulation	Mouse	[18]
		Reduced WIPI2 protein levels and WIPI2 phosphorylation	Mouse	[14]
		Oxidation of ATG3 and ATG7 leading to intermolecular disulfide formation between ATG3 and ATG7	Rat, Mouse	[19]
		Dysregulation of PKA activity resulting in aberrant LC3B/ATG8 S12 phosphorylation	Human	[20]
2	Cargo sequestration	Reduction in p62/SQSTM1/Ref(2)P transcription	Mouse, *Drosophila*	[21,22]
		Cholesterol accumulation disrupts mitochondrial OPTN translocation to prevent mitochondrial clearance	Human, Mouse	[23]
3	Autophagosome closure and autophagosome–lysosome fusion	Decreased translation or increased turnover of Vps4	Yeast	[24]
		Reduced RILP expression to prevent perinuclear localization of autophagosomes	Human	[25]
		Decrease protein levels of Rab2/RAB2A, Arl8/ARL8B, and Rab7/RAB7A	*Drosophila*	[26]
		Decreased interaction between dynein and dynactin	Monkey	[27,28]
4	Autophagic cargo degradation and autophagic lysosome reformation	Lysosomal pH increased with age	Yeast, *C. elegans*	[4,29,30]
		Cathepsin activity decreased via declining lysosomal acidity	Rat, *C. elegans*	[4,31]
		Transcriptional decrease in V-ATPase components	Yeast	[24]
